# A combined Far-FTIR, FTIR Spectromicroscopy, and DFT Study of the Effect of DNA Binding on the [4Fe4S] Cluster Site in EndoIII

**DOI:** 10.1038/s41598-020-58531-4

**Published:** 2020-02-06

**Authors:** Ayaz Hassan, Lucyano J. A. Macedo, João C. P. de Souza, Filipe C. D. A. Lima, Frank N. Crespilho

**Affiliations:** 1 São Carlos Institute of Chemistry, University of São Paulo, 13560-970 São Paulo, Brazil; 2 Goiano Federal Institute of Education, Science and Technology, Campus Rio Verde, 75901-970 Goiás, Brazil; 3 Federal Institute of Education, Science, and Technology of São Paulo, Campus Matão, 15991-502 São Paulo, Brazil

**Keywords:** Chemistry, Physical chemistry

## Abstract

Endonuclease III (EndoIII) is a DNA glycosylase that contains the [4Fe4S] cluster, which is essential for the protein to bind to damaged DNA in a process called base excision repair (BER). Here we propose that the change in the covalency of Fe–S bonds of the [4Fe4S] cluster caused by double-stranded (ds)-DNA binding is accompanied by a change in their strength, which is due to alterations of the electronic structure of the cluster. Micro-FTIR spectroscopy in the mid-IR region and FTIR spectroscopy in the far IR (450 and 300 cm^−1^) were used independently to study the structural changes in EndoIII and the behavior of the [4Fe4S] cluster it contains, in the native form and upon its binding to ds-DNA. Structural changes in the DNA itself were also examined. The characteristics vibrational modes, corresponding to Fe–S (sulfide) and Fe–S (thiolate) bonds were identified in the cluster through far IR spectroscopy as well through quantum chemistry calculations. Based on the experimental results, these vibrational modes shift in their spectral positions caused by negatively charged DNA in the vicinity of the cluster. Modifications of the Fe–S bond lengths upon DNA binding, both of the Fe–S (sulfide) and Fe–S (thiolate) bonds in the [4Fe4S] cluster of EndoIII are responsible for the stabilization of the cluster towards higher oxidation state (3+), and hence its redox communication along the ds-DNA helix.

## Introduction

Metalloproteins containing Fe–S clusters constitute an important group due to several important functions including electron transfer, H_2_ evolution, regulation, and catalysis^1,2^. In particular, there are proteins actively involved in the base excision repair (BER) pathway, interacting with DNA for the search of oxidized/damaged bases and its consequently excising them from the genome^3–5^. The *E. coli* DNA glycosylases EndoIII and MutY are homologues and are involved in the removal of damaged pyrimidines and the removal of mismatched adenine from the A:8-oxoguanine (8-oxoG) pair, respectively^6,7^. Among the six different superfamilies of DNA glycosylases, EndoIII, a bifunctional DNA glycosylase, was the first-discovered enzyme. It belongs to one of the two superfamilies, called helix-hairpin-helix (HhH), which contain Fe–S clusters^7,8^. This name was assigned on the basis of the secondary structural element present in these enzymes that is essential for DNA binding^9^. Other members of this superfamily include MutY, Endonuclease III-like protein 1 (hNTHL1), human MutY homolog DNA glycosylase (MUTYH), and 3-methyladenine glycosylase II (AlkA)^10,11^. Earlier characterization revealed that both EndoIII and MutY contain [4Fe4S] clusters^12^; initially, however, it was not easy to show that the cluster is redox active under physiological conditions, and hence it was basically assigned a structural role^13^. But the quest to prove that these DNA glycosylases detect a single damaged base within an enormous number of normal bases and the specific role of Fe–S clusters in these processes has led to several important revelations about the mechanism behind the signaling between the protein and DNA, and the way in which the former reaches the target site on the latter. It has been shown that though the [4Fe4S] cluster does not play a key role in the structural integrity of the protein, it is essential for the binding of the protein to the substrate^14^.

Upon binding to the DNA, it is now known that the [4Fe4S] cluster stays activated towards oxidation/reduction, whereas it was previously presumed to be redox inactive^4^. Furthermore, a change in its binding affinity towards DNA has been observed with a change in the oxidation state of the [4Fe4S] cluster. For example, a more recent study revealed that the [4Fe4S] cluster in the 2+ oxidation state binds less tightly to DNA as compared to when it is in its 3+ oxidation state^15^. DNA binding not only affects the cluster redox activity, but its charge transfer ability facilitates the redox co-factor in damage detection, as this second property is attenuated due to the presence of a single impairment in the base pairs^16^. In the DNA-charge-transfer (DNA CT) model proposed by Barton and co-workers^5,17^, DNA-CT is considered as a fundamental step in recognizing the damage, as it identifies the repair proteins in the vicinity of the lesion. DNA-mediated CT begins once EndoIII/MutY with their [4Fe4S] cluster in the 2+ oxidation state is oxidized by some cellular oxidant, such as a guanine radical, and this is considered a prerequisite for initiation of the CT process. Due to a greater affinity of the oxidized cluster to ds-DNA, the protein stays bound to the genome, unless it is reduced by a distally-bound protein molecule in the 2+ state, thus decreasing its affinity towards DNA, when it finally detaches and diffuses away. In the absence of DNA damage, the process of scanning the genome through DNA-mediated CT continues, with the CT between oxidized and reduced proteins occurring over long-range distances. Nevertheless, the presence of the lesion in the DNA duplex impedes the signals between the repair proteins; consequently, both the oxidized and reduced proteins remain bound to the duplex and progressively search for the lesion, subsequently repairing it. In the DNA-CT model, these proteins use single-electron transfer to signal to each other across the genome in the search for damage. This model has an important premise: the electron transfer must be reversible and the couple [4Fe4S]^2+/3+^ is kinetically dependent upon DNA-protein binding. DNA-mediated signaling between the reduced and oxidized proteins has also been shown through cross experiments, where DNA processing enzymes with different repair pathways, such as DinG (a DNA helicase that repair R-loop caused by invasion of DNA duplexes by nascent mRNA strands at transcription bubbles in *E*-coli) and EndoIII, in oxidized and reduced state, respectively were mixed together and were found that they work collaboratively for the search and preferentially bind to DNA duplexes with a mismatch in the base pairs^15^.

DNA binding is a key factor not only for activating the [4Fe4S] cluster but for tuning the redox potential^5,18^. EndoIII and MutY have been used in protein film voltammetry on graphite and gold electrodes to measure the redox potential of the [4Fe4S]^3+/2+^ couple in the presence and absence of ds-DNA. It was observed that the binding of DNA is the dominant factor in tuning the redox potential of the cluster in these enzymes, thus shifting its redox potential to a more physiological range ca. ~65 mV vs normal hydrogen electrode (NHE) when EndoIII is bound to DNA, as compared to 130 mV vs NHE for free EndoIII, helping to explain the role of DNA binding in finding a single lesion in the genome^19^. Even though EndoIII electrochemistry was decisive in proving that DNA stabilizes the oxidized form of the Fe–S cluster, [4Fe4S]^3+^, even more recently, the origin of this effect was observed spectroscopically; sulfur K-edge X-ray Absorption Spectroscopy (XAS) was used to study the [4Fe4S] clusters in EndoIII and MutY and evaluate the effects of DNA binding and solvation on the Fe–S bond covalencies^20^.

Solomon and co-workers reported that the presence of DNA in the proximity of the [4Fe4S] cluster destabilized the S 3p orbital energy and increased the covalency (α^2^) of the Fe–S bond by increasing the S 3p character in the metal 3d antibonding orbital (Ψ*). This increase in the Fe–S bond covalency stabilizes the oxidized state of the cluster more than the reduced state, decreasing the redox potential as previously mentioned. This result is unprecedented: for the first time it was shown that perturbations in the vicinity of [4Fe4S] cluster affect the Fe–S chemical bond. This is an important aspect that had been missing from the puzzle of how the interaction between DNA and EndoIII occurs at the molecular level. The result also predicts that the Fe–S bond properties change with the change in redox state of the cluster, due to DNA binding.

With strong evidence of the increased covalency of the metallic cluster, it is expected that the distances and energies of the Fe–S bonds change when the protein interacts with DNA. However, there are not yet any reported infrared vibrational spectroscopy results; shifts in Fe–S bonds vibrational modes are expected for these clusters since these modes are directly dependent on the bond strengths, which are presumed to alter after the interaction with DNA takes place. Far infrared (FIR) spectroscopy (below 500 cm^−1^) is a technique to elucidate the structural properties of compounds whose bonds vibrate at low frequency (energy); in particular, in the structural study of inorganic complexes, information about metal-ligand bond vibrations may be obtained^21^. Therefore, this technique has received great attention in recent years as a probe of the redox co-factor of Fe–S cluster-containing metalloenzymes due to the rich chemical information contained in these metal centers^22^.

Studies on the electronic and structural properties of iron clusters often combine experimental and theoretical techniques^23–27^. For example, Mitra *et al*.^24^ have performed a detailed vibrational study through ^57^Fe-NRVS technique of the [4Fe4S] cluster of the Nitrogenase protein in oxidized, reduced, and all-ferrous oxidation states. Supporting DFT calculations facilitated a much-desired understanding of the [4Fe4S] clusters at the electronic level. However, each protein has its own specificities, and hence a careful theoretical study is needed in each case. To the best of our knowledge, there are no theoretical FTIR spectroscopic studies in the literature on the [4Fe4S] cluster of EndoIII in its native form. Most importantly, it is predicted that the contact between the cluster and DNA is preceded by a pre-interaction step between the polypeptide matrix and the DNA double helix, which should be observable through changes in the active vibrational modes associated with these structures in the mid-infrared (MIR) region. Thus the application of far-IR, which provides direct information on the properties of metal-ligand vibrations, such as the strength of Fe–S bonds, interaction formed within protein and its combination with the mid-IR, where the polypeptide backbone of the protein and the DNA double helix show their vibrational contribution, can thus provide an insight to the structure of isolated EndoIII as well as EndoIII/ds-DNA complex.

**Figure 1:**
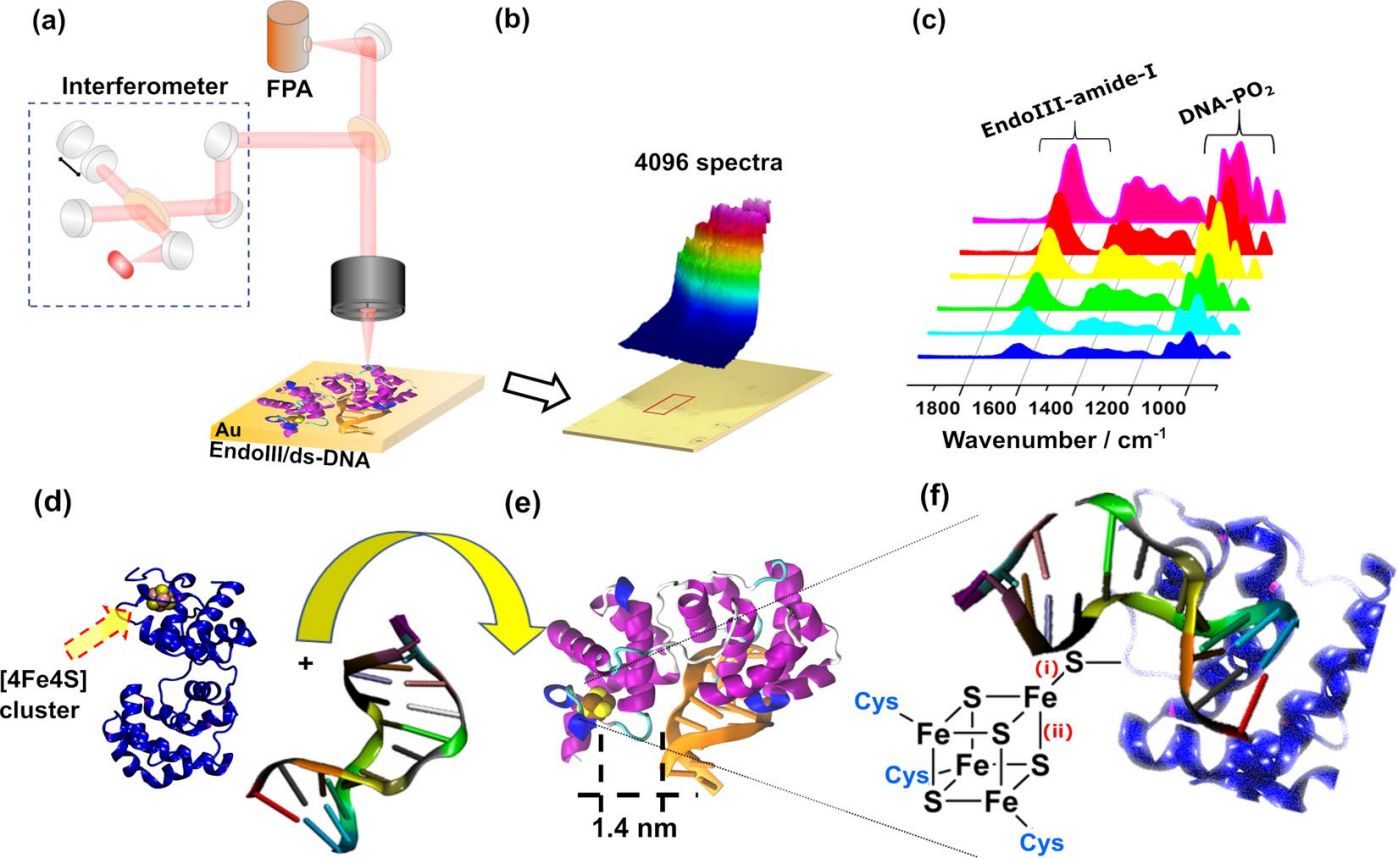
Schematic cartoon showing (**a**) FTIR microscope equipped with focal plane array (FPA) detector; (**b**) The extraction of 4096 spectra from the EndoIII/ds-DNA sample with the help of FPA detector, within an area of 175 × 175 µm^2^, immobilized on a gold-coated glass mirror; (**c**) an array of spectra extracted from the scanned area of the sample showing a regular increase in the intensity of the peaks from outside to inside region of the sample film; (**d**) the binding between the EndoIII (2ABK)^10^ containing the [4Fe4S] cluster and ds-DNA; (**e**) formation of an EndoIII/ds-DNA complex (**1**P59)^28^, with the shortest binding distance of 1.4 nm between the [4Fe4S] cluster and DNA double helix (**f**) the binding between DNA and the protein, which is manifested by changes in the Fe–S bond lengths, marked (i) and (ii), corresponding to Fe–S (thiolate) and Fe–S (sulfide) bonds of the [4Fe4S] cluster, respectively.

Herein, we probe, first, through micro-FTIR spectroscopy, the expected changes associated with the interaction of EndoIII and ds-DNA in the MIR region, specifically, observing the amide-I band of the protein and the band arising from vibrations of the phosphate backbone group of the DNA (Fig. 1a–c). This technique is able to detect small spectral changes because of the large number of spectra that may be collected simultaneously from the microscopically adjacent areas of the sample, a result that has statistical significance in biological assays. Next in the FIR region we focus on the vibrational modes changes of the Fe–S bonds of the [4Fe4S] cluster of EndoIII after its interaction with ds-DNA (Fig. 1d–f). We were able to provide information regarding the structural integrity of EndoIII and DNA and the [4Fe4S] cluster, since effects of the interaction are expected not only in the bulk, but this interaction could bring about changes in the Fe–S bond strength via altering the location of the [4Fe4S] cluster, ultimately leading to shifts in the Fe–S bond stretching frequencies. The measured changes in the Fe–S bond lengths, which are in close agreement with those already reported by the crystallographic data are then correlated with change in the covalency of Fe-S bonds, responsible for the charge transfer properties of the cluster, when ds-DNA is ligated to the protein.

## Results and Discussion

### The origin of the molecular interaction between EndoIII and ds-DNA

**Figure 2:**
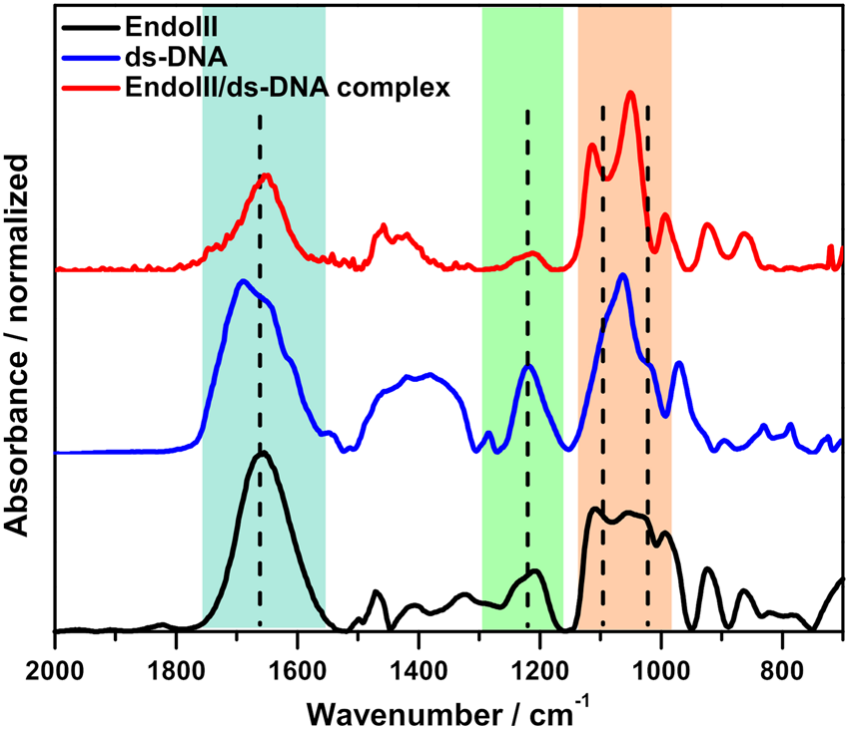
Normalized FTIR spectra in the mid-IR region of (black line) EndoIII, (blue line) ds-DNA, and (red line) the EndoIII/ds-DNA complex, recorded in the transflectance mode. The samples used were in the form of thin films immobilized on a gold-coated glass substrate. Regions are highlighted to show the changes in the amide-I band of the protein and in the phosphate groups of the DNA.

Vibrational spectroscopy has a great potential in the structural characterization of proteins and DNA, particularly in the determination of protein secondary structure, protein conformation, and ds-DNA conformational analysis, because infrared spectra in the mid-IR region exhibit the defining features of both protein and ds-DNA, mostly in the wavenumber range 1700–1000 cm^−1^. Micro-FTIR spectroscopy with a FPA detector was used strategically here because it can produce multiplex chemical imaging of the sample on the gold mirror surface, an easy-to-handle substrate that can support a very small amount of sample, for instance 2 µL of 4 µM of EndoIII/ds-DNA. A thin film of EndoIII/ds-DNA was formed by the drop-casting method. Based on crystal structure (PDB: 1P59)^28^, ds-DNA is thought to interact with EndoIII primarily through weak bonds (non-covalent interactions), thus the changes in the spectrum are expected to be subtle. In order to make our results reproducible and improve the accuracy of our method for detecting small spectral changes, about 100 spectra were averaged for each spectrum, recorded at 4096 different points of the sample (Fig. 1b). FTIR imaging allowed us to spectroscopically examine EndoIII and ds-DNA with a lateral resolution that was only limited by the wavelength-dependent diffraction of the IR light, with a pixel resolution of about 2.5 µm in the reflectance mode, thus making it possible to work with a minute amount of sample^29,30^. The FTIR imaging data results are stored in 3D files and the chemical images of the EndoIII/ds-DNA may be displayed in two and three dimensions in various color scales. Figure 2 shows typical MIR spectra for EndoIII, ds-DNA, and the EndoIII/ds-DNA complex; the spectra plotted are weighted averages of 409600 spectra in each case. Assignments for the different vibrational bands are shown in Supplementary Table S1. For the spectrum of ds-DNA, characteristic IR absorption peaks can be observed at 1690, 1647, 1213, and 1060 cm^−1^ corresponding to the C=O stretching mode of thymine, overlapping stretching mode of the C=O groups of thymine and cytosine, PO_2_ asymmetric stretching of the phosphate backbone, and deoxyribose C–O stretching, respectively^31–33^. Further, the vibrational stretching modes at 967 and 830 cm^−1^ indicate the main S-type sugar marker of DNA^34^. Whereas, the presence of the strong band at 1653 cm^−1^, which corresponds to the amide-I and an intermediate band at 1417 cm^−1^ that is indicative of amide III, in addition to a weak band at 1238 cm^−1^, arise due to C–N stretching, representing the polypeptide structure of the protein^35,36^. Several changes are apparent in the infrared spectrum of the EndoIII/ds-DNA complex, compared to the individual ds-DNA and EndoIII spectra, providing an evidence for the binding of the protein to the DNA molecule.

Although, the spectrum of the EndoIII/ds-DNA complex exhibits features from both protein and DNA, it is observed that the vibrational bands experience some shifts during the binding process. Two of the most prominent changes occur in the regions corresponding to the amide-I band (1653 cm^−1^) and phosphate stretching bands (1050–1220 cm^−1^), as highlighted in Fig. 2. For the amide-I peak, a decrease in intensity and a downshift of 3 cm^−1^ is observed for the complex, in accordance with the literature^32^, where a shift of about 4 cm^−1^ to a lower wavenumber value was reported for the interaction of human serum albumin (HSA) with DNA. We observed the effects of the complex interaction via spectral changes in the EndoIII/ds-DNA spectrum, where the main carbonyl absorption band at 1690 cm^−1^, and the asymmetric and symmetric PO_2_ stretching bands at 1213 and 1085 cm^−1^ in the free-DNA spectrum, all disappeared after complexation with protein. These observation are consistent with the results for DNA and HSA interaction^32^, in which the phosphate group of the DNA backbone is perturbed and the peak is downshifted during the binding process; the same results was reported for R.EcoRII and DNA interaction^31^, though in this case, subtle changes were observed because of the few phosphate molecules available for interaction with the enzyme. Dev and Walters^37^ studied the interaction between the model polypeptide and calf thymus DNA through FTIR spectroscopy and observed that during complex formation, significant changes occur in the region of the phosphate backbone group of  DNA and in the amide-I region of the protein, as we also observe in the present study. They observed the PO_2_ asymmetric stretch only very weakly in the spectrum of the complex. These alterations are possibly due to the attachment of the sugar PO_2_ backbone group to the cysteine residues of the protein through the formation of a hydrogen bond or alternatively via electrostatic attraction; the latter hypothesis is considered to be the general mechanism for DNA-protein interactions, as supported by the work reported by Bruner *et al*.^38^, in which they established an electrostatic interaction between the positively charged helix dipole of EndoIII and the negatively charged phosphate backbone of the DNA strand, with the interaction being intrinsically intimate as compared to that of other DNA glycosylases.

**Figure 3:**
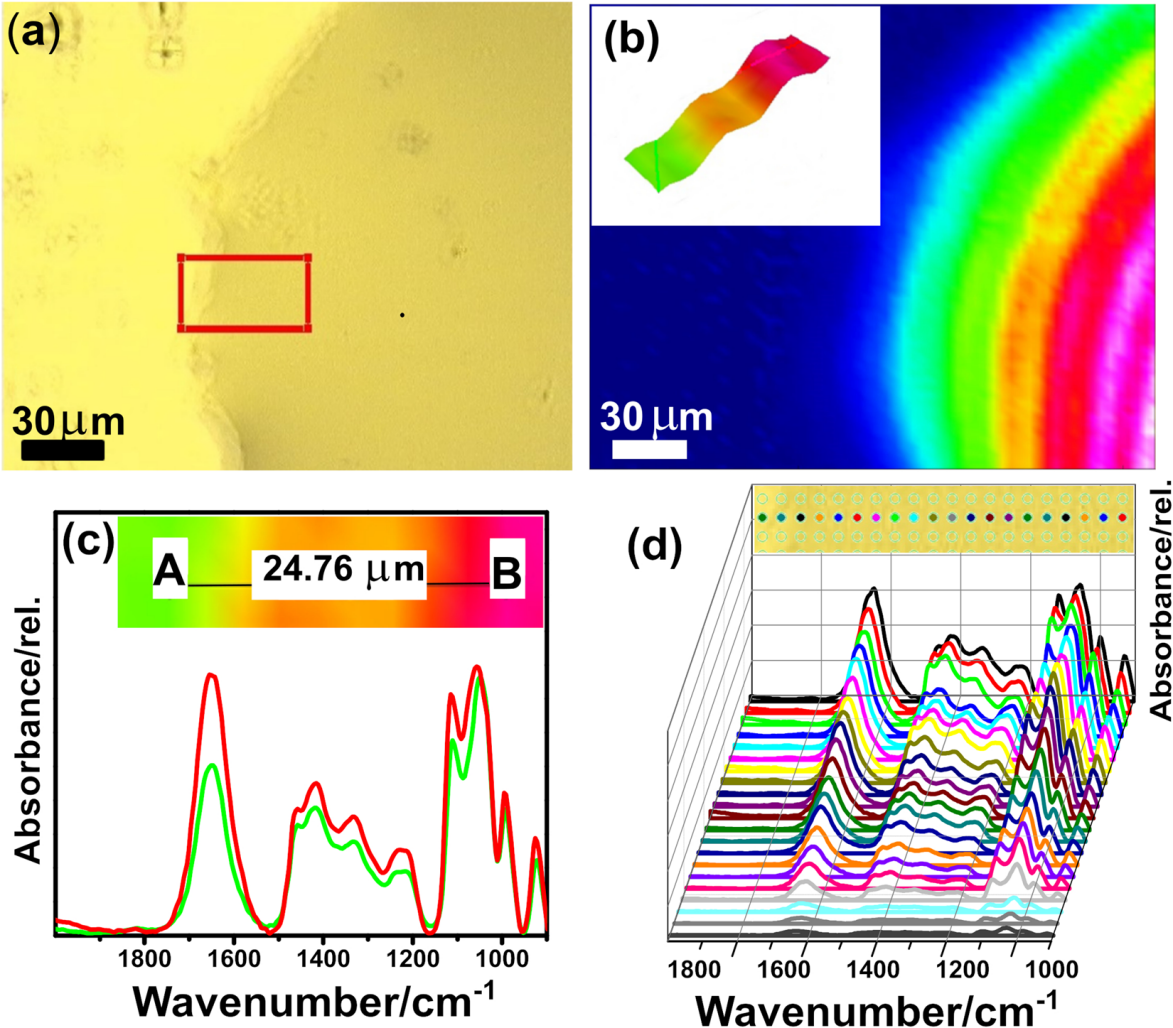
Micro-FTIR spectroscopy of the EndoIII/ds-DNA complex (**a**) Image showing the distribution of the EndoIII/ds-DNA complex film immobilized on a gold-coated glass substrate. The area marked in red is selected for 3D imaging and extraction of spectra; (**b**) chemical image showing the distribution of the amide-I spectral band intensity with an inset showing a 3D image of the red-highlighted area in (**a**–**c**) spectra selected from two different regions A and B of the sample, located as shown in the inset; (**d**) Array of spectra extracted from several pixels of the image.

Next, we discuss the analysis of the chemical images obtained through micro-FTIR chemical imaging. Figure 3 depicts spectra and images of the EndoIII/ds-DNA complex, whereas the results for EndoIII alone are shown in the Supplementary Fig. S1. An optical image is shown in Fig. 3a of the thin sample film on the gold mirror. It is pertinent to mention here that unlike the analysis performed in the FIR region, as will be discussed latter in detail, the sample film is completely dried, thus avoiding possible spectral interference from water, which absorbs in the amide-I region, that can occur when working with proteins in solutions in the MIR region. The chemical image corresponding to the optical image is shown in Fig. 3b, which was obtained by integrating the amide-I band centered at 1653 cm^−1^, and the resultant colors show its distribution within the domain of the sample. A 3D chemical image (see the inset of Fig. 3b) represents the sample area highlighted in red in Fig. 3a.

**Figure 4:**
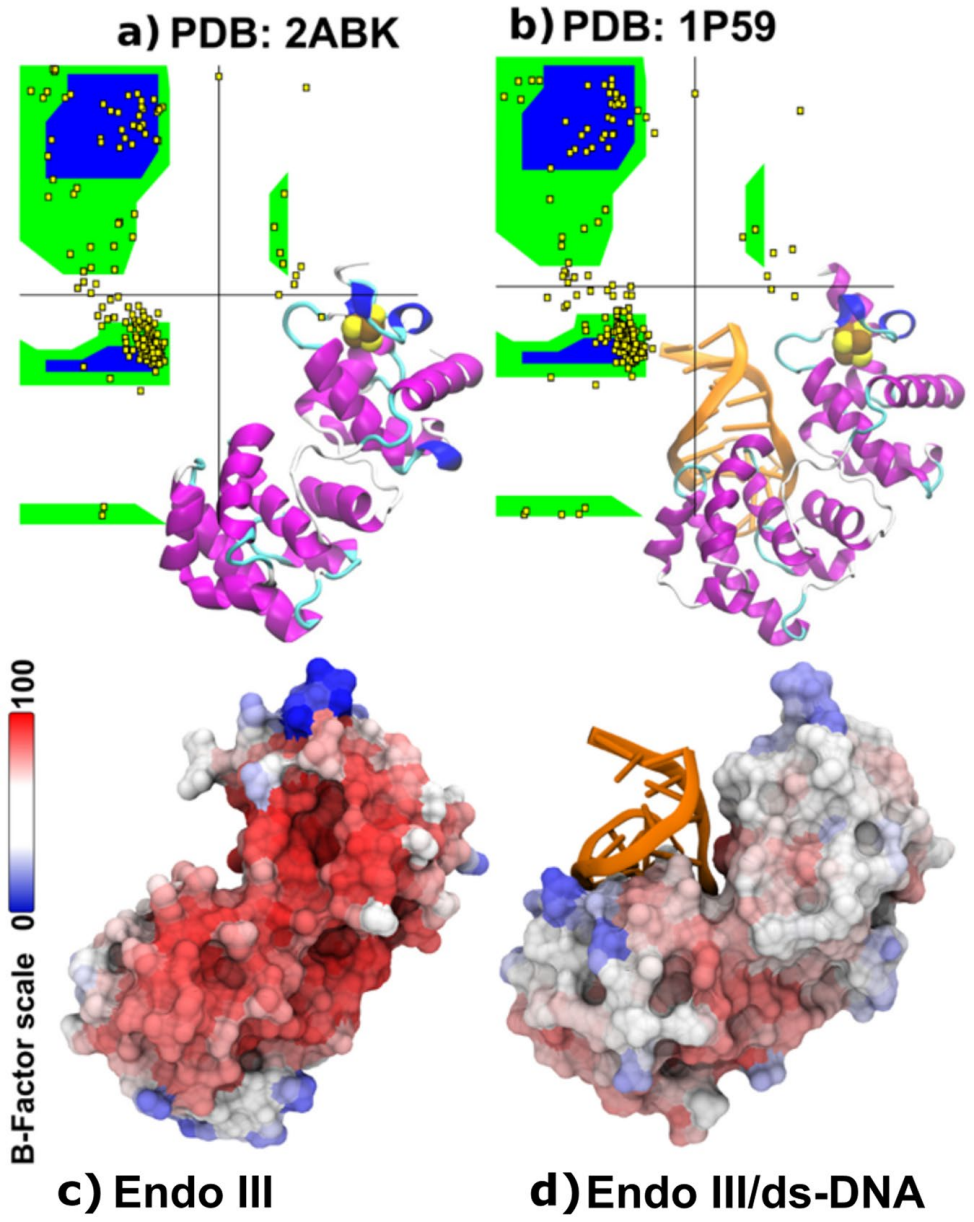
Ramachandran plot for (**a**) EndoIII; (**b**) EndoIII/ds-DNA. For (**a**,**b**), the vertical and horizontal axes refer to the backbone dihedral angle (φ) and the amino acid bond angle (ϕ), respectively. The inset images show ribbon representations of PDB structures, 2ABK^10^ and 1P59^28^, and the colors indicate: ds-DNA (orange); α-Helix (purple and blue); undefined secondary structure (white and cyan); [4Fe4S]^2+^ (brown and yellow balls); Surface representation of (**c**) EndoIII (2ABK) and (**d**) EndoIII/ds-DNA (1P59); the color code indicates the Β−factors obtained for the PDB structures.

The high contrast obtained for the sample distribution between the boundary area and within the film is shown spectroscopically by collecting the spectra from these two different regions (A and B), separated by a distance of about 24.8 µm (Fig. 3c). Since micro-FTIR provides several hundred spectra from a single image, with an effective pixel size of 2.5 × 2.5 µm^2^, and with each pixel consisting of an entire spectrum, some of these spectra of individual pixels are shown (Fig. 3d) by linearly extracting them from the selected region shown in the inset of Fig. 3c. With statistics of several hundred spectra collected, it is possible to accurately verify small structural changes, such as the interaction of the phosphate groups of DNA and a small shift in the amide-I band, as we have seen previously. Once we understood that the amide-I band is an important marker the for EndoIII/ds-DNA complex, Ramachandran plot analysis was carried out, as presented in Fig. 4a–b. These results show a theoretical quantitative analysis of the secondary structure conformation for each protein data bank (PDB) structure. The EndoIII (2ABK)^10^ structure consists of 59% α-Helices, 25% β-Sheets, and 17% undefined secondary structure. On the other hand, EndoIII/ds-DNA (1P59)^28^ has an ordered structure with 62%, 20%, and 18% as the proportions of α-Helices, β-Sheets, and undefined secondary structure, respectively. The B-factor average calculated values are 23.72 ± 11.51 Å^2^ and 38.37 ± 9.67 Å^2^ for 2ABK^10^ and 1P59^28^, respectively (Fig. 4c–d). These structural analyses show that the interaction between EndoIII and DNA results in a more rigid system, and this result may have a direct impact on the analysis of the vibrational properties. Our results allow us to note some interesting trends between the spectroscopic results and the crystallographic data. The vibrational modes of EndoIII are shifted to lower wavenumbers in comparison with the EndoIII coupled with ds-DNA. We also observed a reduction in the degree of freedom observed from the B Factor and the Ramachandran analysis results, caused by DNA stabilization, the complex being less susceptible to temperature, and requiring slightly less energy to activate its vibrational modes.

### DNA binding effect on the [4Fe4S] cluster

**Figure 5:**
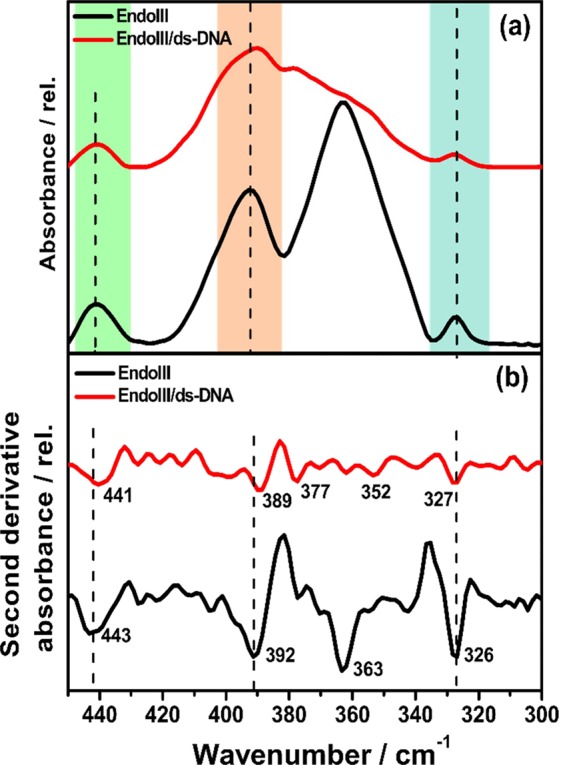
Vibrational changes in the [4Fe4S] cluster. (**a**) FIR spectra of aqueous solutions of (black line) isolated EndoIII and of (red line) the EndoIII/ds-DNA complex; (**b**) second derivative of the spectra depicted in (**a**), highlighting the changes in the absorption peaks, both for Fe–S^t^ and Fe–S^b^.

The [4Fe4S] cluster containing EndoIII has a cubane-like geometry with alternating Fe and S atoms at each corner of the structure, creating the Fe–S bridges (Fe–S^b^), marked by (ii) in Fig. 1f. Inside the enzyme structure, this cluster has four cysteine residues, each coordinated to one iron atoms, the terminal Fe–S (Fe–S^t^) bonds in this case (marked by (i) in Fig. 1f). Both Fe–S^b^ and Fe–S^t^ possess vibrational activity in the FIR region, and FIR spectra of EndoIII recorded in the presence and absence of ds-DNA are shown in Fig. 5a. The spectra shown here correspond to the Fe–S stretching region, appearing within the fingerprint vibrational region; whereas the spectra over the entire FIR region are given in the Supplementary Fig. S2. Unlike ds-DNA and buffer solution (see Supplementary Figs. S2 and S3), which shows no distinguished peaks in their FIR spectra, four signature peaks at 443, 392, 363, and 326 cm^−1^ are observed in the spectrum of EndoIII, which are characteristic vibrational modes of Fe–S bonds (both sulfide and thiolate) in the [4Fe4S] cluster of the redox protein. These vibrational signals confirm that the cluster is in fully active form and is not degraded during the sample preparation step. The vibrational assignments of the different peaks are given in Table 1. The absorption centered at 363 cm^−1^ is the most intense peak in the spectrum and has been assigned to the terminal mode the of Fe–S bond stretch ν(Fe–S^t^), whereas the 326 cm^−1^ peak is the least intense, and has been attributed to the bridging breathing modes of the Fe–S bond ν(Fe– S^b^). Two other bands at 392 and at 443 cm^−1^ appeared with intermediate intensity and are assigned as Fe–S^t^ modes. The highest intensity band at 363 cm^−1^ is consistent with the large dipole moment of the Fe–S^t^ bond produced due to its stronger force constant, which is in turn imposed by the cysteine residues of the polypeptide environment, as compared to the weaker strength of the Fe–S^b^ bond arising from the [4Fe4S] cluster core. Similar vibrational modes have been observed in the IR spectrum of (Et_4_N)_2_[Fe_4_S_4_(SCH_2_Ph)_4_], a synthetic analogue of the [4Fe4S] cluster containing a protein, previously studied for comparative analysis of symmetry distortion in high potential iron proteins (HiPIP) and ferredoxins (Fds) and the IR spectrum of *C. pasteurianum* ferredoxin^22,39^. Resonance Raman spectroscopy, as a commonly used approach to probe low-frequency vibrational modes, has extensively been used for studying the activities of metallic centers in the metalloproteins, including ferredoxins^40–42^. The two most closely related resonance Raman (RR) spectroscopic data, that we found in the literature are those involving EndoIII and PsaC, the last one being a bacterial-like dicluster ferredoxin, where similar bands are observed, though with different relative intensities due to the difference in selection rules of these two different vibrational spectroscopic techniques. The most intense band at 337 cm^−1^ was attributed to the ν(Fe–S^b^) mode, which is thought to be the 326 cm^−1^ band appeared here, with least intensity^7,43^ and is also assigned to the bridging mode. Similarly, bands at 359 and 366 cm^−1^ with intermediate intensities were assigned to the ν(Fe–S^t^) mode in resonance Raman spectra, which corroborates the assignment of the IR band observed here at 363 cm^−1^, with the highest intensity, to ν(Fe–S^t^) mode. Quite similar observations may be made about the 392 cm^−1^ peak that we see, whose complementary peak appear either at 390 or 391 cm^−1^ in these previously reported data and have been assigned to the ν(Fe–S^t^) mode.

**Table 1:** Fe–S Bond Vibrational Mode Assignments.

Experimental (cm^−1^)		DFT (cm^−1^) EndoIII	Assignment
Endo III	EndoIII/ds-DNA		
326	327	331	Fe–S^b^ mode
363	352	343	Fe–S^t^ mode
	377	371	Fe–S^t^ mode
—	389	389	Fe–S^t^/Fe–S^b^ mode
392	—	—	Fe–S^t^ mode
443	441	434	Fe–S^t^ mode

^b^Bridging mode: cluster vibrational mode is active; ^t^terminal mode: the Fe–S (Cysteine) mode is active.

The binding of the DNA to the protein brings some associated changes in the spectrum of the resultant EndoIII/ds-DNA complex, as can be seen by the decrease in the relative intensity of the peaks. Moreover, as well as the main Fe–S^t^ mode at 363 cm^−1^ disappearing, all other vibrational modes are perturbed. Using a mathematical approach to minimize the matrix suppression and maximize the visualization of overlapped absorption peaks^44^, we calculated the second derivatives of the FIR spectra of both EndoIII and the EndoIII/ds-DNA complex (Fig. 5b). These second-derivative spectra exhibit features of the isolated EndoIII structure, and as complexed with ds-DNA, in addition to various low intensity spectral features, which are considered as spectral noise. This approach allows a clearer visualization of the peak positions after the interaction of the protein and ds-DNA.

Although the pattern of the peaks observed in the spectrum of the EndoIII/ds-DNA complex remain similar to those in the free-EndoIII spectrum (Fig. 5a), it is observed that the peaks attributed to the ν(Fe–S^t^) modes at 443 and 392 cm^−1^ in the isolated EndoIII downshift to 441 and 389 cm^−1^ in the EndoIII/ds-DNA complex, respectively. Both these shifts occur in the same direction towards lower wavenumber values. The stabilization based on these results is about 0.036 kJ mol^−1^, as calculated from the shifts in the vibration modes (Fe–S^t^) and reveals the fact that the reorganization of the enzyme structure after interaction with ds-DNA makes the Fe–S (thiolate) bonds stretched, energetically stabilizing the [4Fe4S] cluster of the enzyme. However, the bridging mode at 326 cm^−1^ experienced an upward shift, though by only a very small amount (1 cm^−1^), which suggests a shortening of the Fe–S bonds in the cluster when DNA approaches towards the protein. The lengthening of the Fe–S^t^ bonds and the contraction of the Fe–S^b^ bonds of EndoIII after interaction with DNA is consistent with X-ray crystallographic data previously calculated for the EndoIII/DNA complex^28^. Besides, Khoury and Hellwing^22^ recorded an increase in the Fe-S^t^ bond lengths of the [4Fe4S] cluster in the reduced state on the basis of the downshifting of the vibrational frequency in the IR spectrum of bacterial Fd. This increase in bond length weakens the Fe-S^t^ bonds, which modifies the geometry of the cluster in 2+ state and make it more possible to change to 3+ state. As also reported by Mitra and co-workers^24^ for nitrogenase enzymes, the lower the oxidation state of the [4Fe4S] cluster, the longer the Fe–S bonds are, which suggests that the elongation of the Fe–S bonds of the cluster in the reduced state allow it to convert to the oxidized state and therefore also redox communications along the DNA chain.

**Figure 6:**
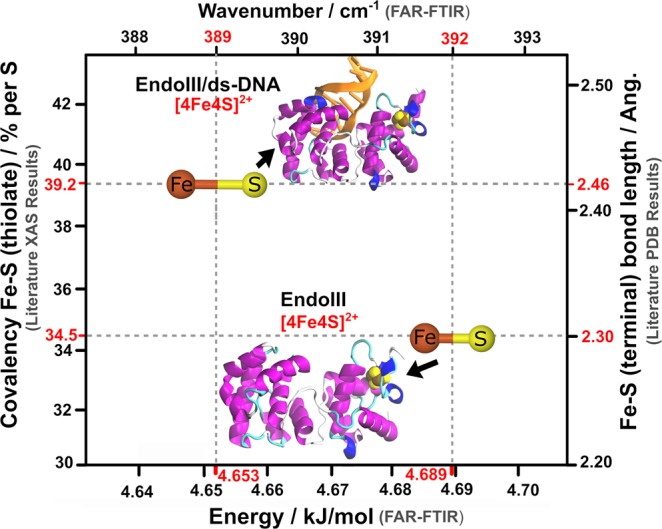
Fe–S bond elongation in terms of increase in covalency and decrease in energy of vibration, when negatively charged ds-DNA approaches close to the [4Fe4S] cluster with 2+ oxidation state, which stabilizes the cluster and energetically makes it more favorable to change to 3+ oxidation state. Literature XAS results are based on ref. ^20^, and literature PDB results are based on ref. ^28^.

Besides the band shifts, when EndoIII is complexed to DNA, the splitting of the 363 cm^−1^ band results in the appearance of two weakly resolved bands at 377 cm^−1^ and 353 cm^−1^, which is confirmed by the second derivative spectra. Thus, the disappearance of one strong peak is concomitant with the appearance of two weak bands in the EndoIII/ds-DNA complex. It is pertinent to mention here that though the binding of the ds-DNA to EndoIII perturbs almost all the main vibrational modes of the [4Fe4S] cluster, these are the ν(Fe–S^t^) vibrations that are affected the most; ν(Fe–S^b^) experiences only some minor shifting. The splitting of one of the ν(Fe–S^t^) modes provides evidence for a conformational change of the protein, whereas the shifting of the other modes provides proof of the stabilization of the [4Fe4S] cluster in its higher oxidation state in terms of bond elongation, as the protein approaches ds-DNA. This stabilization of the oxidized state rather than the reduced state of the [4Fe4S] cluster upon its interaction with ds-DNA can also be correlated to the work recently reported by Solomon and co-workers^20^ (see Fig. 6), where a 1% increase in the total covalency of S causes the redox potential of the cluster to decrease by 3.3 mV, thus a total increase of 58% in the covalency, when DNA binds to EndoIII, makes the redox potential shift by approximately −190 mV, which turns on the functioning of the DNA glycosylase for damage detection. Though initially redox-inert, the binding of DNA introduces negative charge to the enzyme, enabling the S atoms to donate more electron density to the Fe atoms, thus increasing the Fe–S bond covalency, which weakens the Fe-S bond, thus stabilizes the oxidized state (3+) more than the reduced state (2+).

Herein, a change of ~1% in the energy required for bond vibration produced almost the same effect when DNA is complexed with EndoIII; whereas, in isolated EndoIII, the Fe–S^t^ absorption bands appeared at higher wavenumber values, suggesting that the bonds are stronger and hence shorter. However, after DNA is bound to the cluster, the absorption modes are shifted to lower wavenumber values, meaning that the Fe–S bonds are weaker and hence longer (see Fig. 6). This increase in the bond lengths stabilizes the native state (2+) of the cluster, which in turn shifts the redox potential of the cluster to a more physiological range, as previously stated. It is pertinent to mention here, that though the Fe–S^t^ bonds elongate after the DNA binding, the Fe–S^b^ bonds shorten, as revealed by the shifting of the bands to higher wavenumbers. Nevertheless, the shifting of the Fe–S^t^ modes to the lower wavenumber values is much more pronounced (3 cm^−1^) as compared to that observed in the Fe– S^b^ modes (1 cm^−1^), suggesting a dominant bond elongation effect. The difference in the degree to which these different bonds are modulated by the presence of DNA is in accordance with the protein data bank (PDB) literature^28^, where the Fe–S^t^ bond length increases by 0.13 Å and the Fe–S^b^ bond length decreases by around 0.05 Å. The reason the bridging modes are affected little compared to the terminal modes may be the presence of the cluster deep inside the polypeptide matrix, as also indicated by the weak intensity of the bands arising from these modes. Indeed, their covalencies are also affected in the same fashion, i.e 4.7% and 3.3% per S increase for Fe–S^t^ and Fe–S^b^, respectively, when DNA is bound to EndoIII in the solution^20^, thus showing the greater impact of the negatively charged ds-DNA on the terminal modes as compared to the bridging modes.

### DFT simulation of the structure of the [4Fe4S] cluster

**Figure 7:**
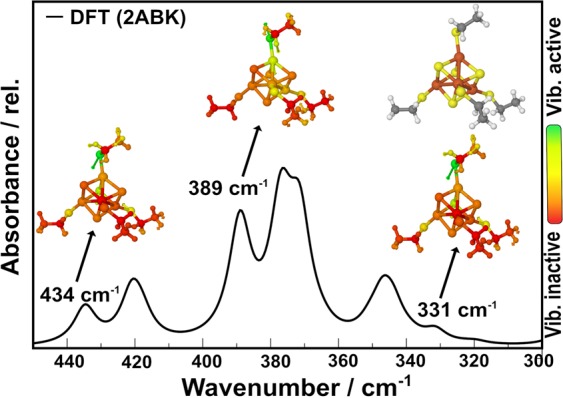
Theoretical IR spectra of the [4Fe4S] cluster using 2ABK starting geometry. Some vibrational modes are shown. Inactive to active regions are presented using color, from red to green, respectively; Atom colors: brown (Fe); yellow (S); blue (N); gray (C); red (O) and white (H).

Theoretical simulations have been made for the [4Fe4S] cluster coordinated to four ethyl thiolates groups and the results, focusing on the principal vibrational modes (Fig. 7), are similar to the experimental results; their corresponding tentative vibrational assignments are provided in Table 1. The theoretical vibrational assignment and spectra in the entire IR region (both mid- and far-IR) are shown in the Supplementary Table S2 and Supplementary Fig. S4, respectively. As expected, Fe–S vibrational frequencies appear only with small intensities below 500 cm^−1^, in close agreement with the experimental data. As can be seen in Fig. 7, the vibrational modes appeared in the theoretical model at almost the same position as was observed experimentally for EndoIII. For example, the bands at 443, 392, 363, and 326 cm^−1^ observed in the samples correspond to the bands noticed at 434, 389, 371, and 331 cm^−1^ in the theoretical model. The difference between the intensities of the bands observed experimentally and those calculated theoretically, as well as a small difference in their positions, may be due to the different nature of the environments involved, since in the former case the [4Fe4S] cluster is bound within a complex protein environment, whereas in the theoretical model, only a few ethyl thiolates groups are present.

Moreover, the Fe–S bond lengths (terminal and bridging) calculated for isolated model (2ABK)^10^ by using geometry that mimic the experiment are shown in the Supplementary Table S3 and the results are compared with the experimental bond lengths calculated through X-ray crystallography for EndoIII^28^. In addition, the results for the PDB code 1P59, which show the changes in bond lengths after the DNA is ligated to the EndoIII are also given. The average values (see Table 2) for the Fe–S^b^ distances are 2.31 ± 0.02 Å and 2.22 ± 0.10 Å for the 2ABK and 1P59 structures, respectively; Fe–S^t^ bonds are 2.3 ± 0.02 Å and 2.46 ± 0.12 Å. These changes in the bond lengths observed in the PDB structures may suggest a possible influence of the DNA presence, since the Fe–S^t^ bonds increase by about 0.13 Å and the Fe–S^b^ bonds shorten by around 0.05 Å. On the other hand, the Fe–S^b^ and Fe–S^t^ bond lengths calculated for isolated EndoIII model are in reasonable agreement with PDB results. On the basis of these results, it can be inferred that the presence of ds-DNA in the vicinity of the [4Fe4S] cluster brings an associated change in the vibrational modes, which in turn reflects the changes in bond lengths. This is an interesting observation, revealing the fact that the alteration of the [4Fe4S] cluster environment reallocates the Fe–S bond frequencies, showing the sensitivity of the electron transfer co-factor, which plays an important role in the search for damaged bases within the vast sea of duplex DNA.

**Table 2:** Fe–S^b^ (Sulfide) and Fe–S^t^ (Thiolate) Bond Lengths.

Mode	EndoIII (2ABK)	EndoIII/ds-DNA (1P59)	DFT (2ABK)	DFT (1P59)
Fe–S^b^	2.31	2.26	2.33	2.32
Fe–S^t^	2.30	2.43	2.26	2.26

All values are averages, in Å, obtained from the PDB codes 2ABK^10^ and 1P59^28^. DFT results are from models using the geometries on 2ABK and 1P59, keeping the α-Carbon of the cysteine ligand fixed.

## Conclusion

In this study, we have evaluated the vibrational properties of the [4Fe4S] cluster and other parts of the EndoIII enzyme, with and without its interaction with ds-DNA, using FTIR spectroscopy in the mid- and far-IR. Micro-FTIR spectroscopy in the mid-IR was able to show the interaction between the protein and the genome, where the principle of the interaction is evidenced in the amide-I region of the spectrum of the enzyme; the phosphate group bands of the DNA spectrum give evidence of a weaker, mainly electrostatic, interaction. By using a focal plane array detector, it was possible to collect several-hundred spectra from the sample, which made possible the detection of small spectral changes to the vibrational modes during the interaction process. Through infrared spectroscopy in the far region, it was possible to identify the IR bands specific to the Fe–S vibrational modes. Four vibrational signatures observed in this region not only indicate the existence of the [4Fe4S] cluster in EndoIII but facilitated correct assignment of the specific modes, whether arising from the cluster or from the cluster and the attached cysteine residues. A perturbation in both the Fe–S^t^ and Fe–S^b^ modes upon interaction between the protein and ds-DNA reveals that Fe–S bond distances are altered during the interaction. The shifting of some of the bands to lower frequencies due to interaction with ds-DNA means that the cluster becomes probably more stable, in terms of the increase in bond lengths towards the occurrence of the cluster in a higher oxidation state (3+) without much energetic stress. Our results are confirmed by DFT calculations, in which similar absorption modes appeared in the [4Fe4S]-(-SC_2_H_5_S)_4_ model, with some minor differences. Our experimental data are in close agreement with PDB results on the fact that perturbing the [4Fe4S] cluster environment induces some structural changes by changing the bond lengths, which stabilizes the cluster in the native state to make possible the change in oxidation state and this, in turn, prepares it for functioning in damage detection in DNA. The results obtained here comprise an important basis for further investigation of the interaction of DNA glycosylases with duplex DNA, and hence help in understanding the mechanism behind damage detection among molecular bases.

## Methods

### Samples preparation

Single-stranded DNAs 5′-GTG AGC TAA CGT GTC AGT AC-3′ (20 ^A^) and 5′-GTA CTG ACA CGT TAG CTC AC-3′ (20 ^T^) were purchased from Integrated DNA Technologies (USA) and stored at −20 °C before use. Double-stranded DNA (ds-DNA) was prepared by mixing 20^A^ and 20^T^ at 1:1 mole ratio with a final concentration of 4 µmol L^−1^ and annealing them by heating to 90 °C followed by slowly cooling them to room temperature. *E. coli* EndoIII was purchased from Sigma Aldrich (≥90%-SDS-Page) and stored at −20 °C before use. A solution of 20 mmol L^−1^ sodium phosphate, pH 7.6, 0.5 mmol L^−1^ EDTA, and 150 mmol L^−1^ NaCl was used for preparing EndoIII sample solutions. A total protein concentration was used in this study instead of cluster loading. The experiments were performed using a previously published data^15^, considering that there is a ratio of 1 unit of EndoIII per DNA strand in the EndoIII/ds-DNA complex.

### Micro-FTIR experiments

Micro-FTIR spectra and chemical images were recorded in the reflectance mode using a spectral resolution of 4 cm^−1^ and 100 scans in the 4000–600 cm^−1^ spectral window using an FTIR spectrometer (Vertex 70 v, Bruker) coupled with an FTIR microscope (Hyperion 3000, Bruker) equipped with liquid N_2_ and cooled 64 × 64 elements focal plane array (FPA) detector. Each element of the FPA works as an individual detector, making possible the measurement of 4096 spectra from different regions of the samples in a single detection with a spatial resolution of 2.5 µm. As a total of 100 scans are used, which means in a single measurement, we have collection of 409600 spectra. The spectrum provided is thus a weight average of the 409600 spectra. Chemical images were obtained by integrating the area under the peak corresponding to a specific vibrational mode in the FTIR spectrum. The measurements were performed by using the samples in the form of thin films on gold-coated glass substrates. The substrates were initially cleaned chemically by washing with acetone and distilled water and then electrochemically using cyclic voltammetry in 0.1 mol L^−1^ H_2_SO_4_ solution for 15 cycles in the potential range of 0.1 to 1.5 V vs Ag/AgCl reference electrode at a scan rate of 0.05 V s^−1^. A thin film was then formed by applying 2 µL of each 4-µmol L^−1^ EndoIII solution (in 20 mmol L^−1^ phosphate buffer, 0.5 mmol L^−1^ EDTA, 150 mmol L^−1^ NaCl, pH 7.4), separately to the substrate, and then incubating overnight at 4 °C. In the case of the EndoIII/ds-DNA complex, equimolar amounts of both EndoIII and ds-DNA were mixed and incubated at 4 °C for 30 min, then applied to the gold-coated glass substrate and left overnight for further incubation. This long incubation time was provided, in order to ensure a maximum possible binding of the protein to the DNA. The use of low temperature for incubation was to avoid the degradation of the cluster within the protein. All the FTIR spectra shown here are difference spectra, where the absorption signals corresponding to the background are subtracted from those of the samples.

### Far-IR experiments

FTIR spectra in the far-IR were acquired using the attenuated total reflection (ATR) mode in a Vertex 70 v (Bruker) instrument by internal reflection in a diamond crystal under vacuum condition. Solutions of ds-DNA and EndoIII were placed directly onto the ATR crystal. The diamond crystal was cleaned with isopropanol and dried before each measurement. EndoIII/ds-DNA complex solution was prepared by mixing initially equal amount of equimolar solutions of both EndoIII and ds-DNA with a final concentration of 2 µmol L^−1^ and then incubated overnight at 4 °C. Difference spectra, where the background signals are subtracted from those of the samples were collected using a room-temperature DLaTGS (deuterated L-alanine doped triglycine sulfate) thermal detector, and the resultant spectra are the average of 32 interferograms of 4 cm^−1^ spectral resolution in the spectral window 700–100 cm^−1^.

### Computational details

The IR spectra were simulated using Density Functional Theory (DFT) as implemented in the Gaussian 09 software^45^. The model employed here used the [4Fe4S]^2+^ cluster with 4 ethyl thiolates [SCH_3_CH_2_] molecules each with a charge of −1 (see Fig. S5, Supporting Information) modeling the cysteines attached to the 4 iron atoms, with a total charge of −2 for the [4Fe4S](SC_2_H_5_)_4_ model. The calculations were carried out, based on the geometry of the protein codes 2ABK^10^, keeping the α-carbons fixed. The basis-set employed was LANL2DZ, and the exchange correlation functional was B3LYP10^46^ and vacuum conditions were used. Both systems had their atomic structure relaxed, using a quadratic-convergence self-consistent field (SCF) procedure^47^. The single resulting theoretical vibrational spectra did not include any negative frequencies, meaning that the obtained calculations were properly converged and relaxed, within the given level of theory. A similar setup to the calculations of the vibrational properties of [xFeyS] clusters were successfully used, as reported in the literature^23–27^. The Jmol software^48^ was used to visualize the results and vibrational modes.

### Computational structural study of the PDB codes

In order to understand the conformational changes of EndoIII in the presence of DNA, we computed the B-Factor and Ramachandran plots for the structures. The B-Factor quantifies the thermal motion of the protein. It is defined as ***B*** = **8π**^**2**^**〈*u***^**2**^**〉**, where **〈*u***^**2**^**〉** is the mean-squared displacement obtained via X-Ray measurements. High (low) B-Factors represent flexible (rigid) structures. Moreover, the Ramachandran plot is a method to visualize protein secondary structure through dihedral angles of the backbone (φ) and the bond angle (ϕ) of the amino acids^49^. These angles allow the determination mainly of α-Helix, 3_10_-Helix and β-Sheet conformations for a given PDB structure^49^. These structural properties of the enzyme were studied using the 2ABK^10^ (resolution 1.85 Å) and 1P59^28^ (resolution 2.50 Å) PDB codes and V.M.D. 1.9.2 software^50^.

## Supplementary information

Supplementary information.
