# Supplementary material for: A combined Far-FTIR, FTIR Spectromicroscopy, and DFT Study of the Effect of DNA Binding on the [4Fe4S] Cluster Site in EndoIII
Source: Sci Rep. 2020 Feb 6;10:1931. doi: 10.1038/s41598-020-58531-4 (PMC7005299; doi:10.1038/s41598-020-58531-4)
Supplement: Supplementary file 1 — Supplementary information. [file 41598_2020_58531_MOESM1_ESM.docx]

**Supplementary Information**

**A combined Far-FTIR, FTIR Spectromicroscopy, and DFT Study of the Effect of DNA Binding on the [4Fe4S] Cluster Site in EndoIII**

Ayaz Hassan^1*^, Lucyano J. A. Macedo^1^, João C. P. de Souza^2^, Filipe C. D. A. Lima^3^, Frank N. Crespilho^1*^

^1^*São Carlos Institute of Chemistry, University of São Paulo, 13560-970 São Paulo, Brazil.*

^2^Goiano Federal Institute of Education, Science and Technology, Campus Rio Verde, 75901-970, Goiás, Brazil.

^3^Federal Institute of Education, Science, and Technology of São Paulo, Campus Matão, 15991-502 São Paulo, Brazil.

Corresponding authors email: [ahasan333@yahoo.com](mailto:ahasan333@yahoo.com), and frankcrespilho@iqsc.usp.br

**Table S1.** Assignments of the vibrational modes observed in the FTIR spectra of isolated EndoIII,

The EndoIII/ds-DNA complex, and isolated ds-DNA in the mid-IR (700 – 2000 cm^-1^).

| Peak (cm^-1^) | EndoIII | ds-DNA | EndoIII/ds-DNA |
| --- | --- | --- | --- |
| 20 |  | CH out-of-plane bending vibrations | Same peak appeared |
| 780 | CH out-of-plane bending vibrations |  | A small peak is observed at 785 |
| 782 |  | Sugar-phosphate vibration | A small peak is observed at 794 |
| 826 |  | Main S-type sugar marker | A small peak is observed at 813 |
| 822 | Unassigned |  | A small peak is observed at 821 |
| 863 | Unassigned |  | A broad peak is observed at 859 |
| 890 |  | Deoxyribose ring vibration | Peak disappeared |
| 967 |  | O–P–O bending | Peak disappeared |
| 924 | Unassigned |  | Same peak appeared |
| 993 | Unassigned |  | Same peak appeared |
| 1016 |  | Unassigned | Peak disappeared |
| 1033 | C=O stretching coupled with C-O bending |  | Peak disappeared |
| 1055 | Unassigned |  | An intense peak appeared at 1050 |
| 1060 |  | Deoxyribose C-O stretching | Peak disappeared |
| 1110 | C–C stretching |  | Peak shifted to 1114 |
| 1213 |  | PO_2_ asymmetric stretching of phosphate backbone | Peak disappeared |
| 1210 | Amide-III; C-H stretching and N-H in plane bending (often with significant vibration from CH_2_ wagging) |  | A broad peak appeared at 1209 |
| 1238 | Amide-III |  | Peak disappeared |
| 1281 |  | N–H deformation | Peak disappeared |
| 1329 | CH_2_ wagging |  | A small peak appeared at 1339 |
| 1376 |  | Stretching C–N, cytosine, guanine | The peak is distorted in to several smaller peaks |
| 1417 | C–N Stretching; N–H deformation; C–H deformation |  | Peak disappeared |
| 1417 |  | C-H deformation | A very minute peak appeared |
| 1455 |  | Extremely weak peak of DNA |  |
| 1457 | CH_3_ asymmetric bending |  | Peak appeared |
| 1511 |  | CH in plane bending | Peak disappeared |
| 1540 |  | Base vibration | Peak disappeared |
| 1606 |  | Adenine vibration | Peak disappeared |
| 1647 |  | C=O overlapping stretching mode of thymine and cytosine; C=N and N–H stretching mode of adenine, thymine, guanine and cytosine | Peak disappeared |
| 1653 | Amide-I |  | Peak shifted to lower wavenumber value |
| 1690 |  | C=O stretching of thymine | Peak disappeared |
| 1817 | Unassigned |  | Peak disappeared |


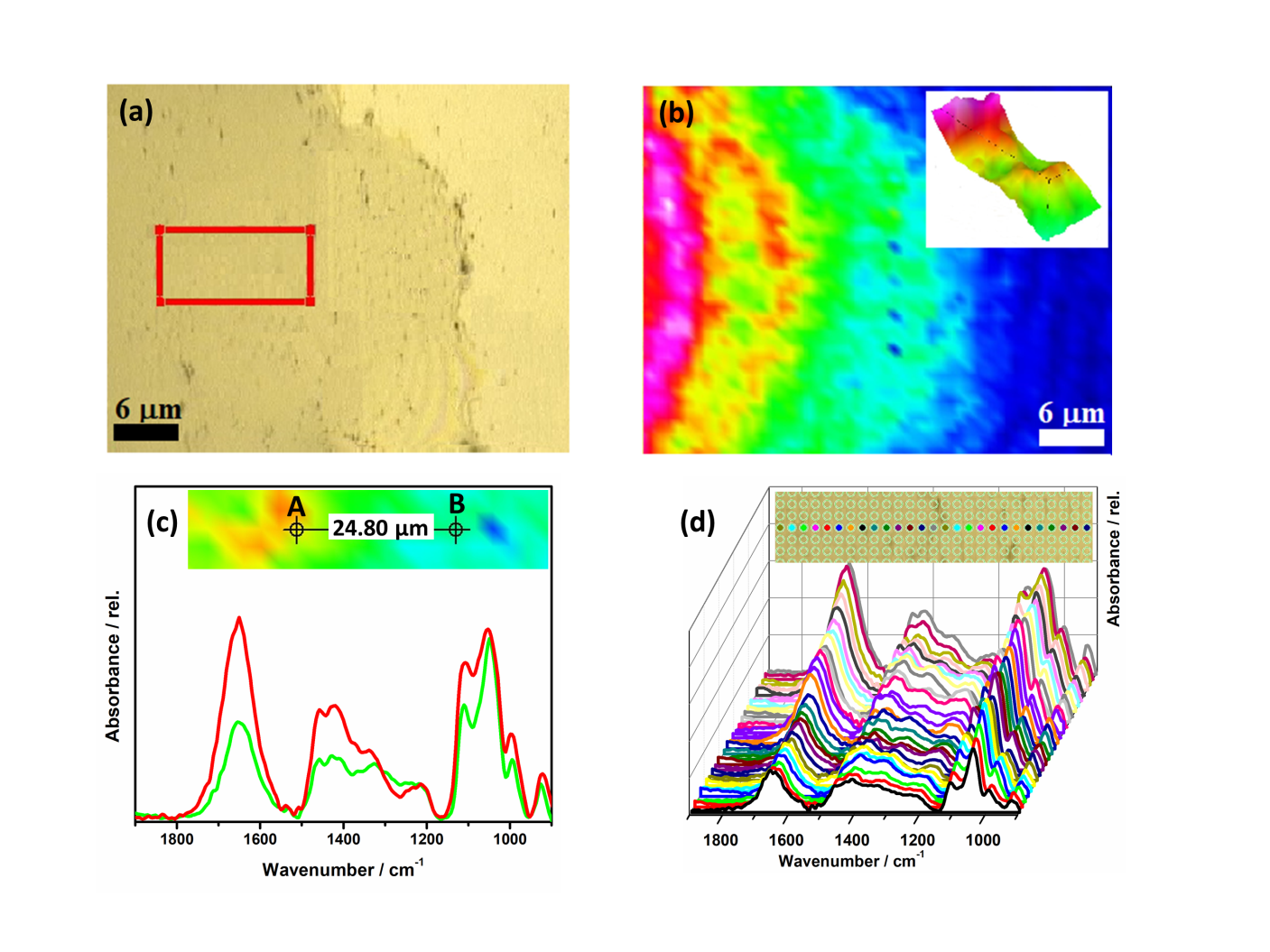


**Figure S1.** Micro-FTIR spectroscopy of EndoIII. (a) Optical image showing the distribution of EndoIII film immobilized on gold coated glass substrate. The area marked in red is selected for 3D imaging and extraction of spectra; (b) chemical image showing the distribution of the amide-I spectral band with the inset showing a 3D image; corresponding to the area highlighted in red in a; (c) spectra selected from two different regions, A and B of the sample marked on the inset; (d) array of spectra extracted from several pixels of the sample film.





**Figure S2.** Far-IR spectra of aqueous solutions of (●) EndoIII, (●) ds-DNA, and (●) EndoIII/ds-DNA covering the entire far-IR region. These spectra were recorded in ATR mode using a diamond crystal. Aqueous solutions of the samples were directly drop-casted on the crystal, and the measurement was performed under vacuum.





**Figure S3.** Far-IR spectrum of the buffer solution composed of sodium phosphate (20 mmol L^-1^, pH 7.6), EDTA (0.5 mmol L^-1^) and NaCl (150 mmol L^-1^).

**Table S2.** Theoretical vibrational assignments for the high-intensity peaks of the model species

[4Fe4S]^2+^-[C_2_H_5_S]_4_^-^ using 2ABK and 1P59 geometry.

| Wavenumber (cm^-1^) | Vibrational modes assignment |
| --- | --- |
| [4Fe4S]^2+^-[C_2_H_5_S]_4_^-^ |  |
| 2ABK Geometry |  |
| 331.42 | Fe-S^b^ |
| 343.88 | Fe-S^t^ |
| 371.40 | Fe-S^t^ |
| 376.92 | Fe-S^t^ / Fe-S^b^ |
| 389.00 | Fe-S^t^/ Fe-S^b^ |
| 420.87 | Fe-S^b^ |
| 434.67 | Fe-S^t^ |
| 1001.4 | CH_3_ wagging. |
| 1214.83 | CH_2_ wagging / CH_3_ twisting. |
| 1216.95 | CH_2_ wagging / CH_3_ twisting. |
| 1423.21 | CH_3_ wagging. |
| 1455.54 | CH_2_ scissoring. |
| 1502.38 | CH_3_ scissoring. |
| 1520.68 | CH_3_ scissoring. |
| 2923.49 | CH_2_ asymmetric stretching. |
| 2968.21 | CH_2_ symmetric stretching. |
| 3001.90 | CH_2_ / CH_3_ symmetric stretching. |
| 3087.03 | CH_3_ asymmetric stretching. |
| 3118.98 | CH_3_ asymmetric stretching. |

^b^bridging mode: the Fe-S stretching mode from the cluster. ^t^terminal mode: Fe-S stretching mode from thiolate-cluster.


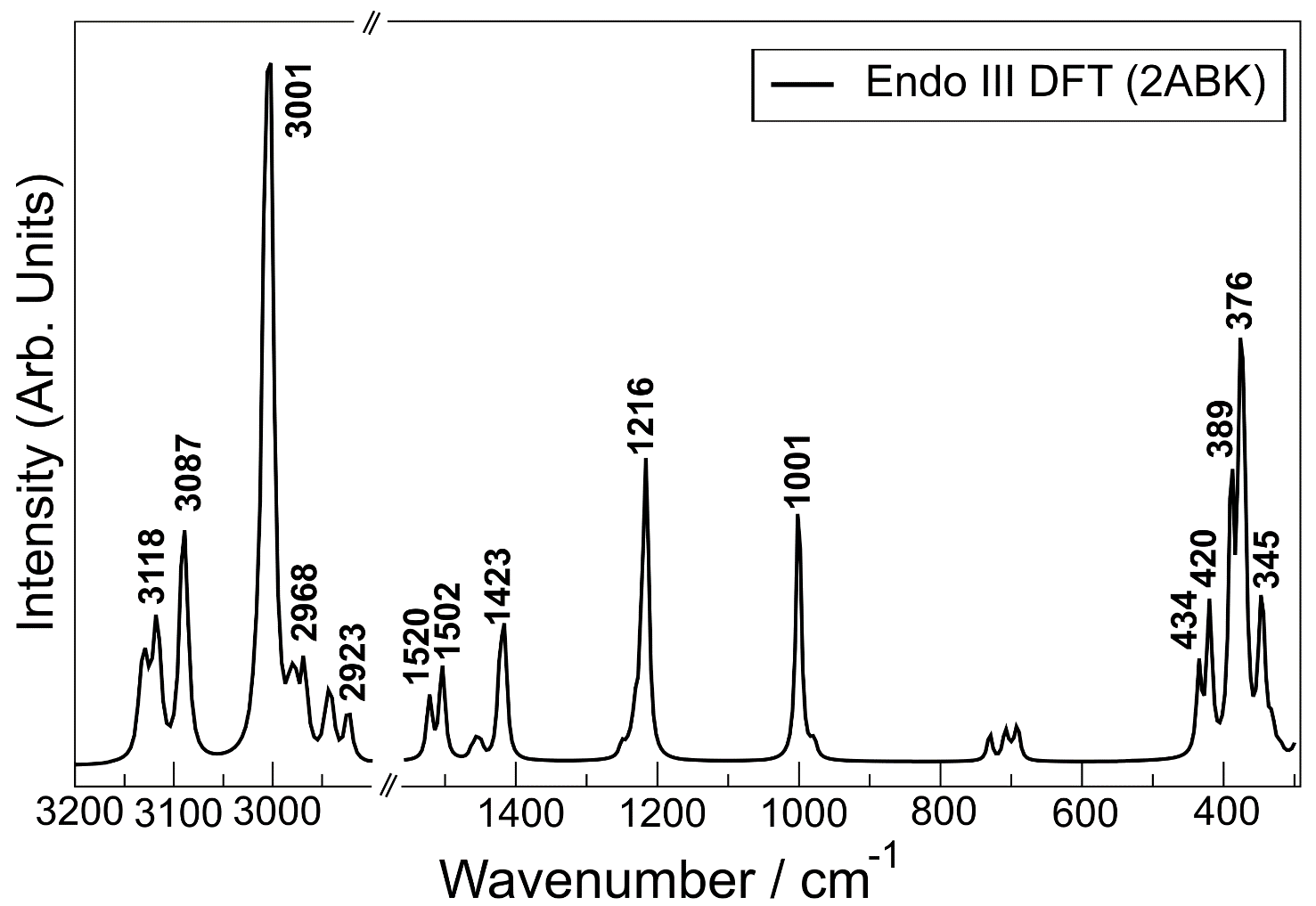


**Figure S4.** Theoretical FTIR spectrum for the [4Fe4S]^2+^-[C_2_H_5_S]_4_ model. The relative intensities are adjusted for a better visualization and the numbers above the peaks represent their respective wavenumbers.

**Table S3:** Fe-S^b^ (sulfide) and Fe-S^t^ (thiolate) bonds lengths, in Å, obtained from the PDB codes 2ABK^7^ and 1P59^8^ and DFT results for the models using the 2ABK geometry, keeping the α-carbon of the cysteine ligand fixed.

| Vibrational mode | Endo III (2ABK) | Endo III/ds-DNA (1P59) | Model 2ABK(Endo III geometry) |
| --- | --- | --- | --- |
| Fe-S^b^ | 2.32 | 2.32 | 2.22 |
|  | 2.29 | 2.30 | 2.23 |
|  | 2.30 | 2.10 | 2.21 |
|  | 2.31 | 2.21 | 2.33 |
|  | 2.32 | 2.18 | 2.41 |
|  | 2.29 | 2.34 | 2.38 |
|  | 2.31 | 2.23 | 2.32 |
|  | 2.31 | 2.15 | 2.32 |
|  | 2.33 | 2.40 | 2.44 |
|  | 2.29 | 2.11 | 2.31 |
|  | 2.28 | 2.26 | 2.42 |
|  | 2.33 | 2.10 | 2.32 |
| Avg | 2.31 | 2.22 | 2.33 |
| Std. Dev | 0.02 | 0.10 | 0.08 |
| Fe-S (Cys) | 2.30 | 2.63 | 2.27 |
|  | 2.33 | 2.42 | 2.22 |
|  | 2.29 | 2.339 | 2.27 |
|  | 2.28 | 2.46 | 2.28 |
| Avg | 2.30 | 2.46 | 2.26 |
| Std. Dev | 0.02 | 0.12 | 0.03 |


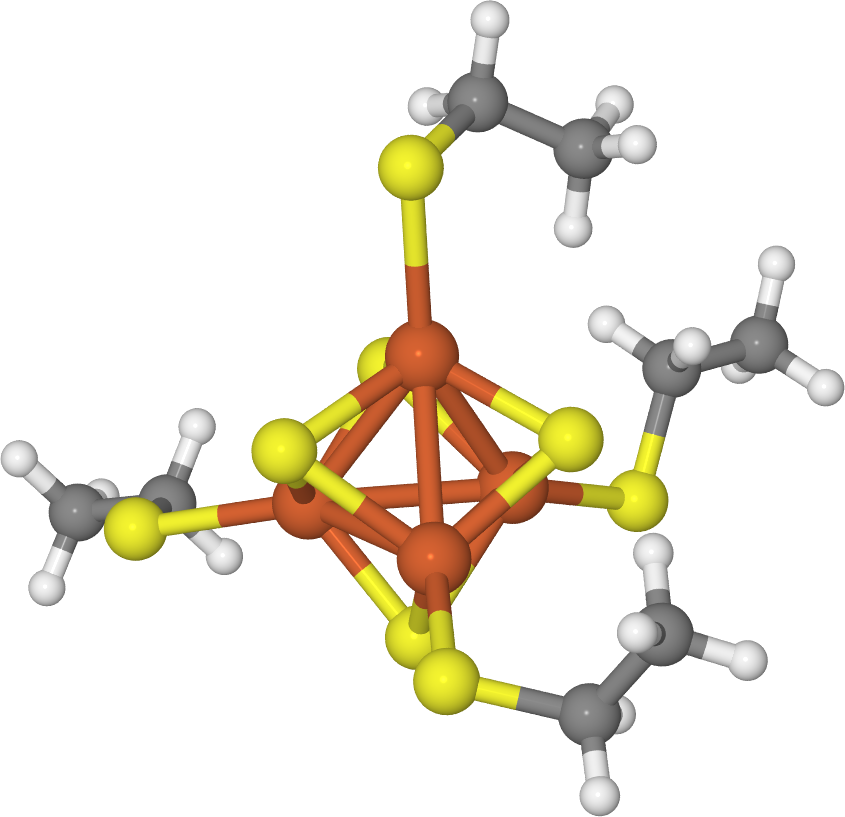


**Figure S5.** Theoretical model [4Fe4S]^2+^ cluster with four ethyl thiolate groups, [CH_3_CH_2_S]^-^, attached. Color key: brown (Fe); yellow (S); gray (C); white (H).
